# Optimisation of a Microwave Synthesis of Silver Nanoparticles by a Quality by Design Approach to Improve SERS Analytical Performances

**DOI:** 10.3390/molecules29143442

**Published:** 2024-07-22

**Authors:** Julie Horne, Pierre Beckers, Pierre-Yves Sacré, Charlotte De Bleye, Pierre Francotte, Nicolas Thelen, Philippe Hubert, Eric Ziemons, Cédric Hubert

**Affiliations:** 1 Laboratory of Pharmaceutical Analytical Chemistry, Department of Pharmacy, CIRM, ViBra-Sante Hub, University of Liege (ULiege), 4000 Liege, Belgium; 2 Research Support Unit in Chemometrics, Department of Pharmacy, CIRM, University of Liege (ULiege), 4000 Liege, Belgium; 3 Laboratory of Medicinal Chemistry, Department of Pharmacy, CIRM, University of Liege (ULiege), 4000 Liege, Belgium; 4 GIGA-Neurosciences, Cell Biology, University of Liege (ULiege), 4000 Liege, Belgium

**Keywords:** surface enhanced Raman scattering (SERS), silver nanoparticle synthesis, process optimisation, Quality by Design (QbD), microwave

## Abstract

A major limitation preventing the use of surface-enhanced Raman scattering (SERS) in routine analyses is the signal variability due to the heterogeneity of metallic nanoparticles used as SERS substrates. This study aimed to robustly optimise a synthesis process of silver nanoparticles to improve the measured SERS signal repeatability and the protocol synthesis repeatability. The process is inspired by a chemical reduction method associated with microwave irradiation to guarantee better controlled and uniform heating. The innovative Quality by Design strategy was implemented to optimise the different parameters of the process. A preliminary investigation design was firstly carried out to evaluate the influence of four parameters selected by means of an Ishikawa diagram. The critical quality attributes were to maximise the intensity of the SERS response and minimise its variance. The reaction time, temperature and stirring speed are critical process parameters. These were optimised using an I-optimal design. A robust operating zone covering the optimal reaction conditions (3.36 min–130 °C–600 rpm) associated with a probability of success was modelled. Validation of this point confirmed the prediction with intra- and inter-batch variabilities of less than 15%. In conclusion, this study successfully optimised silver nanoparticles by a rapid, low cost and simple technique enhancing the quantitative perspectives of SERS.

## 1. Introduction

During recent decades, surface-enhanced Raman scattering (SERS) has experienced a considerable expansion across the world. This can be explained by the numerous advantages of this vibrational technique. Indeed, SERS conserves the Raman spectroscopy benefits such as the specificity and the real-time or in vivo analysis while getting around its major limitations [1,2,3]. Therefore, the Raman signal can be enhanced up to a factor 10^9^ on average, which enables the overcoming of fluorescence and avoidance of interference [4,5]. This sensitivity improvement is possible because the analyte of interest is adsorbed or close to the metallic nanostructures that act as antennae to amplify the Raman scattering from the analyte. Most of the time, these nanostructures, also called SERS substrates, are nanoparticles made of gold (AuNps) or silver (AgNps) [6,7].

SERS can be employed to realize qualitative and quantitative studies in various domains [8,9,10,11]. However, the latter are underdeveloped due to, in particular, the lack of synthesis repeatability [12,13,14,15]. The signal exaltation is directly impacted by the SERS substrates properties, such as their nature, size or shape, but it is also influenced by the analyte itself [16,17]. The use of a repeatable SERS substrate synthesis protocol is essential to measure a repeatable SERS signal and thus to improve the quantitative perspectives of this promising technique [18]. Commercial substrates are available. They are more homogeneous but are very expensive, and their dispersing matrix composition is generally confidential and can lead to interferences with biological or pharmaceutical samples. This is the reason why many researchers work to produce suitable SERS substrates, but their protocols remain generally expensive and time-consuming [19]. The most widely used synthesis process has remained unchanged for many years: a chemical reduction of a metallic salt described by Lee and Meisel [20]. In the case of AgNps, the reaction consists of reducing silver nitrate by trisodium citrate, added in excess and acting also as a stabilising agent to avoid nanoparticle aggregation [21]. The obtained SERS substrates are in the form of a nanoparticle suspension. However, the resulting Nps present various sizes and different shapes. Previous works tried to optimise the synthesis of AgNps by the Lee and Meisel protocol since some important parameters such as the stirring speed and the speed flow of citrate addition are not described in the original manuscript [22]. Unfortunately, some parameters remained difficult to control, such as the uniformity of the heating or the atmosphere of the reaction, limiting the optimisation process.

The present study aims to present an innovative strategy to robustly optimise a rapid, low-cost, easy to implement and repeatable synthesis process to obtain AgNps [23]. This robust protocol optimisation could lead to an improvement in the repeatability of the Nps’ properties from batch to batch but especially an improvement in the protocol reproducibility from laboratory to laboratory. Robust syntheses and analysis protocols are needed to improve the scientific reproducibility and consistency. Silver was selected as a starting point considering its widespread use as a SERS substrate and its high exaltation factor. In this study, microwave irradiation was considered to guarantee better control and a uniform heating during the process. Moreover, in comparison with the classical chemical reduction methods, microwave synthesis enables a fast formation of AgNps that are more homogeneous, with a higher yield and a lower price [24,25,26,27].

With this aim, the Quality by Design (QbD) strategy was considered in this study to robustly optimise the synthesis process. This approach consists of the realisation of several successive designs of experiments (DoEs), generating a maximum of information with a minimum of experiments [28]. In practice, several factors were concurrently modified to consider their interaction. Finally, a method operable design region (MODR) showing the accomplishment of the objectives according to the different parameters is modelled [29]. Consequently, the QbD strategy is applied for the optimisation of a microwave method for the synthesis of homogeneous AgNps in a repeatable way to generate reproducible SERS signals. This approach can also be repeated for other synthesis processes.

## 2. Results and Discussion

**Figure 1:**
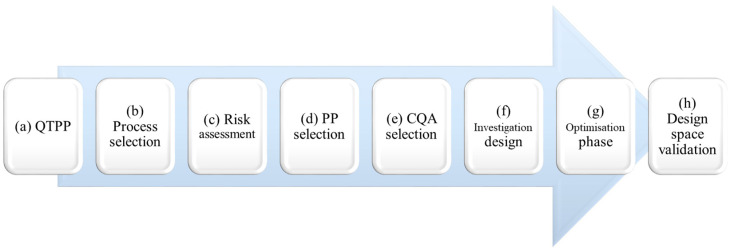
Quality by Design strategy developed in the present study (QTPP: Quality target product profile, PP: Process parameter, CQA: Critical quality attribute).

The Quality by Design strategy was used to optimise the AgNps synthesis using microwave irradiation. This strategy is summarised in Figure 1 and the following steps:

(a) The objective of this study can be summarised by the quality target product profile (QTPP).

(b) Then, the process to achieve this goal needs to be selected.

(c–e) Thereafter, a risk assessment will help to determine the critical quality attributes (CQAs) and the process parameters (PPs). While the CQAs are the criteria to evaluate the quality of the response, the PPs are the parameters giving the response required by the CQAs.

(f) Indeed, these PPs will be added to a DoE (generally a screening design) to evaluate if they have a critical impact (CPPs) on the CQAs.

(g) Further, only CPPs will be considered for the optimisation design. This second type of DoE allows a better estimation of the impact of the CPPs.

(h) Finally, a design space showing robustness zones can be plotted. This zone displays the optimal values for each CPP with the probability of success for the announced aim. In the objective to validate this optimal zone, the synthesis conditions will be tested many times.

Table S1 groups together the Quality by Design abbreviations and a short explanation of each term. Concerning the continuous improvement, this strategy needs to be recurrently evaluated to guarantee its quality for a long time [30,31]. Finally, the performance of the optimised process was compared to that of the classical Lee-Meisel synthesis.

### 2.1. QTPP and Process Selection

In this strategy, the first step consists of defining goals such as the main objective of the study or the process to be optimised. The goal is represented by the quality target product profile (QTPP) and consists to optimise the microwave synthesis of AgNps to enable broad quantitative applications of SERS in a rapid, low cost, repeatable and easy to implement manner. In this way, repeatable SERS substrates need to be obtained both in terms of size and shape. The repeatability was then preferred to the sensitivity.

The reduction of silver nitrate by trisodium citrate with the help of microwave irradiation appeared to be a promising alternative to the classical and widely used Lee–Meisel reduction [32]. Microwave synthesis is straightforward to execute, significantly shortens both the preparation and reaction and also offers advantageous features, including improved temperature control and more uniform heating [33]. This phenomenon can be explained by creating more homogeneous Np cores thanks to a more harmonious heating [34]. Effectively, waves can enter deeply and quickly into the sample [24,33].

### 2.2. Risk Assessment, PP and CQA Selection

**Figure 2:**
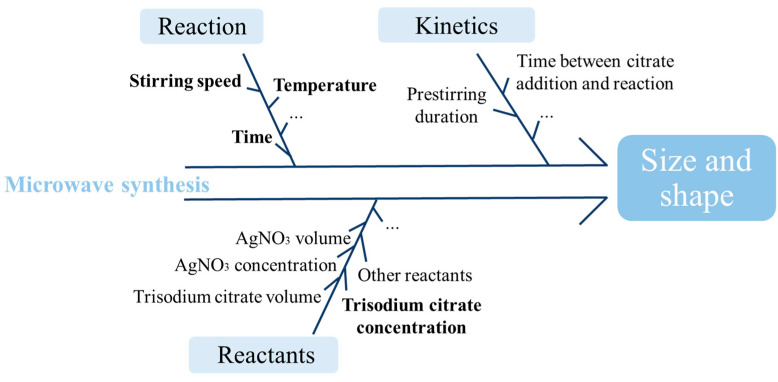
Ishikawa diagram used to determine the process parameters for the optimisation of the microwave synthesis of silver nanoparticles.

For the objective of selecting the different process parameters (PPs) of which the criticality must be evaluated, a risk assessment was considered. Among the different tools, the Ishikawa technique was retained. The filled diagram is presented in Figure 2 with the retained PPs in bold.

Based on the results obtained during previous work and a review of the literature, the trisodium citrate concentration seemed to directly impact the formation of AgNps [22,33,35]. Given that citrate is the only reactant added to the metallic salt solution and that it plays the role of both a reducing and stabilising agent, it appeared essential to consider its concentration in the DoE. The reaction time and the temperature may also have a non-negligible impact on the homogeneity of the synthesised Nps [35,36]. Preliminary studies have shown a strong correlation between these two parameters and the formation of AgNps that then needs to be considered for the elaboration of the design. The investigated parameters’ values were constrained following Equation (1) to ensure that the DoE is performed with experimental conditions leading to the formation of AgNps. Low temperatures combined to low reaction times were insufficient to generate Nps.

(1) 7.69×time+temperature ≥157.69

The impact of the stirring speed was also evaluated taking into account that it may influence the size and shape of Nps during their formation and may also avoid the generation of aggregates [37,38]. A large interval was considered, according to the instrument specifications.

Concerning the kinetics of the reaction, the impact of pre-stirring was explored during preliminary studies but was not found to be significant. An influence of the time between the addition of the reducing agent and the start of the reaction could not be excluded. However, the risk was mitigated by maintaining a 40 s interval between the introduction of trisodium citrate solution to the AgNO_3_ solution and the initiation of the reaction by the microwave.

Four parameters were selected as continuous variables for the investigation design. These PPs are the trisodium citrate concentration, the reaction time, the reaction temperature and the stirring speed. The PPs with their intervals are presented in Table 1.

The critical quality attributes (CQAs) are the observed responses leading to the predefined goal. Given that the shape and size of the AgNps directly impact the SERS response, the intensity and the relative standard deviation (RSD) of SERS measurements were selected. Indeed, heterogeneous suspension induces a variable signal between analyses. In the optimisation design, a SERS intensity greater than 30,000 counts was targeted, with a maximum RSD of 15% being acceptable.

### 2.3. Investigation Design

After the determination of all the parameters, a first DoE consisting of an investigation design was drawn. The A-Optimal design was selected to reduce the average variance of the parameter estimates, enabling us to detect significant parameters [39]. The synthesis conditions are presented in Table S2. After the synthesis of the different batches, SERS analyses were performed by transmission to analyse a more representative sample volume.

Preliminary tests were realised to optimise the analysis conditions. A crystal violet concentration of 0.75 × 10^−6^ M was retained because this value is a good compromise to obtain a good signal and avoid detector saturation for most of the syntheses. Moreover, the TRS configuration was also optimised to be suitable for a maximum of synthesis conditions. Concerning the investigated results, the signal intensities varied from 169 to 55,458 counts, while the RSDs (n = 9) were between 1 and 35%. Figure S1a shows the SERS spectra to illustrate the differences between the syntheses. The nature and concentration of the aggregation agent was not included in the PPs because these factors should be optimised for each analyte and matrix.

**Figure 3:**
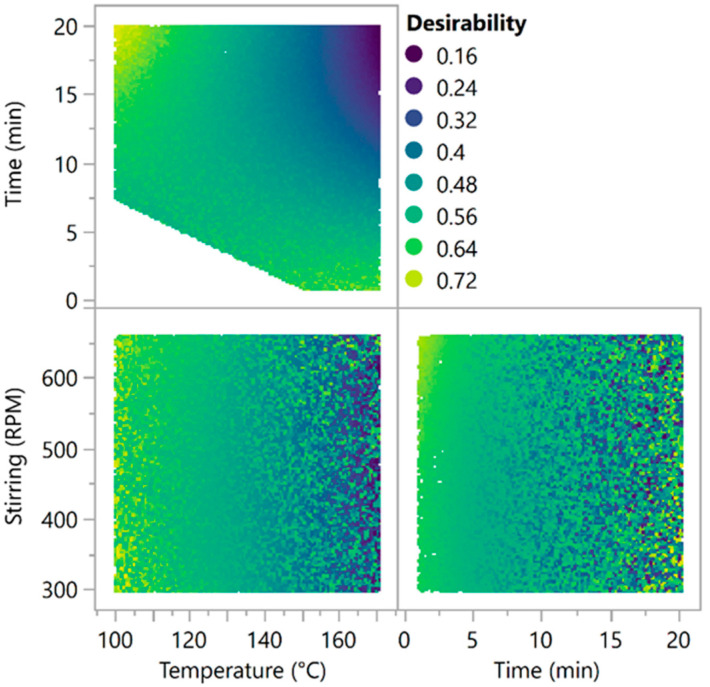
Simulated conditions with the regression models of the investigation design. The dots are coloured based on the function of the log transformed SERS signal intensity and the RSD. Yellow conditions denote high intensity; blue conditions denote low signal intensity.

To explore and interpret the design, a log transformation was applied to the SERS intensity to ensure positive predictions, a more linear relationship and a restricted range of values. The log(intensity) and the response RSD were modelled with a linear mixed model. Based on the results of stepwise modelling, the more important parameters are the temperature and time main effects, the time quadratic effect and the time × temperature interaction. Regarding the signal RSD, the selected factors were the temperature, reaction time and stirring main effects; the time and stirring quadratic effects; and the interactions time × speed and time × stirring. No model found a significant impact of the citrate concentration. The optimisation design was therefore performed on the selected CPPs: reaction time, temperature and stirring speed. Figure 3 represents the computed overall desirability over the whole experimental space for the selected CPPs. The design is an irregular pentagon due to the constraint described in Equation (1). The shift in colouration towards yellow indicates higher desirability, meaning conditions of high signal intensity and low RSD. Two areas seemed to be interesting to explore. The first one concerns the long reaction time with a low temperature, and the second is the short reaction times with a more elevated temperature. The stirring speed seemed to have a very weak impact on the overall desirability. Among the two interesting areas, the faster one was selected for the optimisation design.

### 2.4. Optimisation Phase

Once the CPPs were identified and selected, they were optimised. An I-optimal design was built to minimise the average prediction variance over the design space. The parameter ranges are described in Table 1. This design was performed thrice and averaged to minimise the SERS variability. After the syntheses of the different AgNps batches, SERS measurements were performed. A kinetic study was added to the initial protocol (three samples for each KCl concentration for each suspension), which consisted of taking a spectrum each minute during 10 min. The signal intensity was obtained in the same way as for the investigation design, and its stability was observed. Generally, a signal increase was observed followed by a plateau. The subsequently analysed intensity was the average signal intensity of the plateau. Even if the stabilisation time of the signal varied from one synthesis to another, the same time interval was kept for the repetitions of the same synthesis. In total, nine measurements were obtained for each condition (three independent syntheses and three independent replicates per synthesis). The reported signal intensity was the average of these nine measurements. This also allowed analysis of the intra- and inter-synthesis variability. To this end, a one-way ANOVA was performed with a random effect on the synthesis factor. Using the decomposed variance, the intra-synthesis RSD and intra- + inter-synthesis RSD were computed and modelled. The optimisation design experiments and the obtained average results are illustrated in Table 2.

Figure S2 shows some pictures obtained by TEM for different AgNps syntheses coming from the first replicate of this design. Substantial heterogeneity in size and shape can be highlighted, justifying the variability in the presented SERS results.

**Figure 4:**
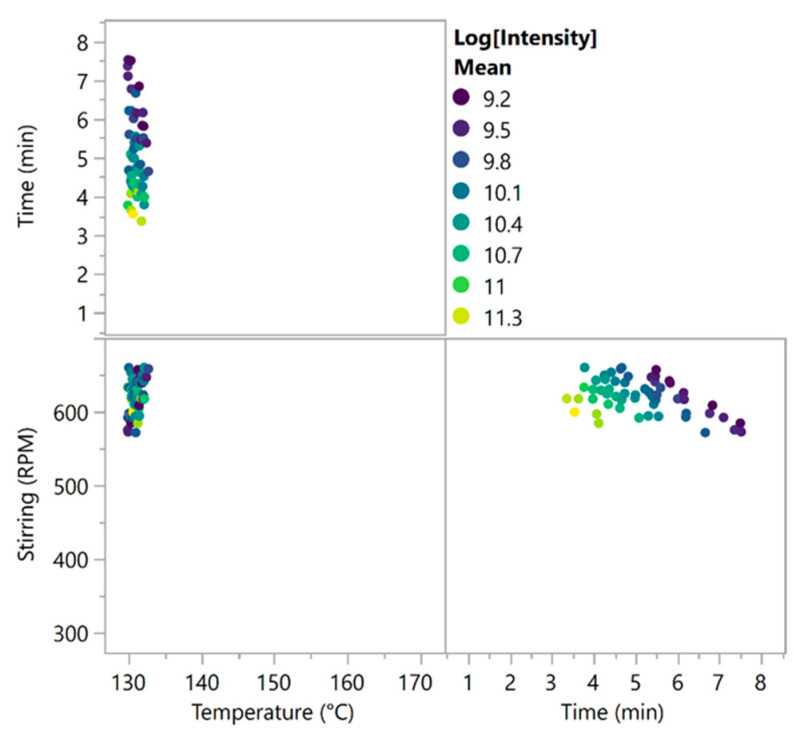
MODR of the optimisation design results with selected conditions matching the requirements (signal intensity > 10,000 counts, variability intra-synthesis < 15% and intra- + inter-synthesis variability < 20%). The dots are coloured by the log of intensity, and the shift in colouration towards yellow indicates a higher SERS signal intensity.

The regression analyses of the different responses allowed for identifying the most repeatable conditions. A simulation experiment was performed, and new conditions covering the experimental range were simulated via a Gaussian process. The results are presented in Supplementary Figures S3–S5. To determine the MODR and the conditions to be tested to validate the design, limits were fixed for each CQA: signal intensity should be higher than 10,000 counts, and the intra- and intra- + inter-synthesis RSDs should be lower than 15% and 20%, respectively. The matching conditions are presented in Figure 4. One can see that a low temperature and high stirring rate were desirable and that the reaction time should vary between 3 and 8 min. However, the signal intensity was higher for short times.

Based on these results, the selected optimal conditions were 3.36 min, 130 °C and 600 rpm. The highest tested stirring speed (660 rpm) was not chosen for the stirring because its effect was more pronounced on the intensity rather than on the RSD, which was not significantly affected. Therefore, a balance in the optimal area was found by selecting a stirring speed of 600 rpm.

### 2.5. Design Space Validation

Based on the optimisation design, the optimal conditions were determined but needed to be validated. Consequently, the selected conditions (3.36 min–130 °C–600 rpm) were used to synthesize six new batches. A complete characterisation of the AgNps was performed to observe the homogeneity and the repeatability of the syntheses.

**Figure 5:**
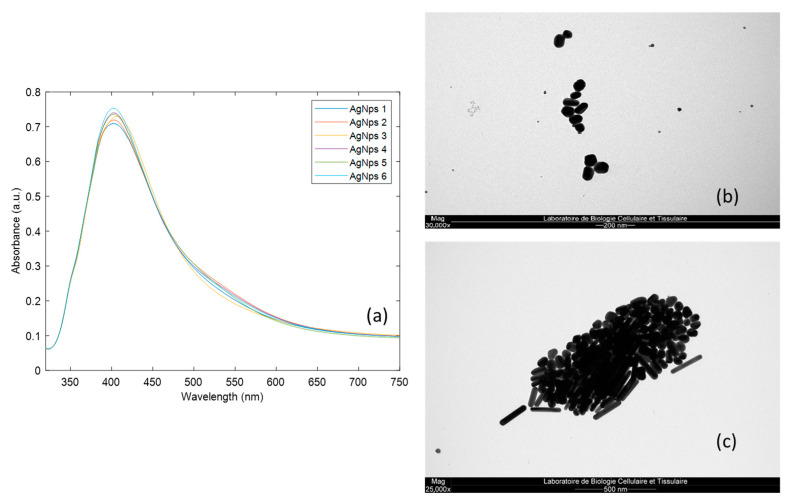
Characterisation of optimised AgNps, (**a**) UV–visible spectra, (**b**,**c**) pictures from transmission electron microscopy for two different syntheses illustrating the presence of two main populations.

First, each synthesis was analysed by a UV–visible spectrophotometer. The observed absorption peak corresponds to the plasmon resonance band that forms the foundation of the SERS phenomenon [40]. These spectra are depicted in Figure 5a. Only minor variations were noted among the different spectra, indicating a good repeatability between the batches [41].

Secondly, dynamic light scattering (DLS) was used to measure the mean size of each synthesis, hypothesising that the AgNps were spherical. Two populations were generally observed, except for the first synthesis, which contained only one population of 81 nm. The five additional syntheses were divided into 73% of 74 nm, and the remaining 27% measured 15 nm, on average. The RSD of the five syntheses for the mean size of AgNps was less than 3.0% for the two populations. On the other hand, the zeta potential reflecting the suspension stability was also measured. For all the syntheses, this value was negative due to the presence of an excess of citrate. In absolute value, the higher the potential, the more stable is the suspension [42]. On average, the syntheses presented a zeta potential of −45 mV with an RSD of 4.4% among the six syntheses.

The shape of the AgNps was observed by transmission electron microscopy (TEM), as shown in Figure 5b,c. The DLS results were confirmed since the presence of two populations was also observed by TEM, which highlighted the coexistence of rods and spheres of different sizes. The TEM results were similar from one synthesis to another. While the presence of rods and spheres can lead to signal variability, each population seems more homogeneous. Moreover, it can be hypothesised that the coexistence of rods and spheres is responsible for a more intense SERS signal.

Finally, SERS analyses were conducted following the same protocol established for the optimisation design. A stable signal was observed after 8 min for each batch. The observed synthesis variability inside a batch was between 7 and 15%, while the batch-to-batch variability presented a value of 9% for a mean SERS signal intensity of 34,191 counts. These values were inside the specifications and in agreement with the modelled performances. The SERS spectra are presented in Figure S1b. The AgNps synthesis process can then be considered as repeatable from batch to batch in terms of size and shape. Moreover, the SERS signal intensity was repeatable both within a suspension and between batches.

### 2.6. Comparison Study

**Figure 6:**
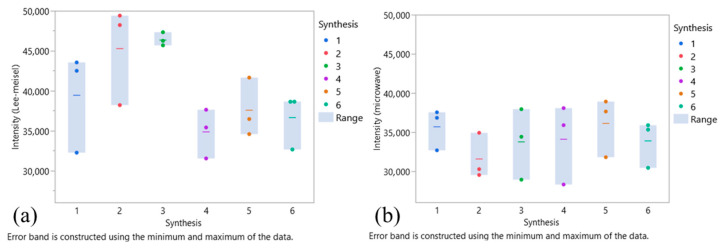
Repeatability of SERS intensity of AgNps batches synthesised by the (**a**) Lee–Meisel or (**b**) microwave-optimised protocol. Each point represents the SERS intensity of a sample, while the coloured line is the average SERS intensity per synthesis.

In order to compare the performances of the microwave-optimised synthesis process with that of the Lee–Meisel protocol, the intra- and inter-batch RSDs were calculated based on six syntheses for each synthesis route. Figure 6 illustrates the performance metrics for each synthesis. The microwave synthesis showed more uniformity in terms of intensity and repeatability. In contrast, while the Lee–Meisel syntheses exhibited higher SERS intensities, they also showed significant variations both within and between the batches. Figure S6 shows TEM pictures of the AgNps synthesised according to the Lee–Meisel protocol. By this process, AgNps of different shapes and multiple sizes are obtained, explicating the variability observed in the SERS measurements.

Intra-synthesis variations were comparable for both the Lee–Meisel and microwave synthesis routes with RSD values of 10.8% and 11.1%, respectively. However, when considering both the intra- and inter-synthesis variabilities, the RSD for the Lee–Meisel synthesis was equal to 14.8%, while the microwave-optimised process demonstrated a lower RSD of 11.1%. Figure S1c compares the SERS spectra for these two synthesis processes. Table S3 shows the different RSD values obtained based on SERS acquisitions for different concentrations of crystal violet. The values calculated demonstrated that the microwave-optimised process improved the repeatability of SERS measurements.

In addition, the Lee–Meisel syntheses showed a zeta potential of −40 mV on average with an RSD of 3.0% among the six batches. The mean size measured by DLS was 70 nm with an RSD of 6.9%, while the microwave syntheses presented an RSD less than 3.0%. Figure S7 compares the UV–visible spectra for both synthesis protocols. Taking into account the full width at half maximum, reflecting the size dispersity, nanoparticles synthesized by the Lee–Meisel protocol were more heterogeneous than those produced by the microwave-optimised protocol. The inter-synthesis dispersity also varied more for the Lee–Meisel syntheses.

This suggests that the optimisation of AgNps synthesis by a microwave technique slightly improves the repeatability of SERS analyses. Additional advantages such as the short reaction time and simplified preparation also render the developed synthesis protocol favourable for SERS quantitative analyses.

Specifically, the Lee–Meisel synthesis, from preparation to the end of the reaction, typically requires half a working day including one hour for the reaction itself. In contrast, the microwave-optimised protocol needs only 3.36 min for the reaction and approximately 30 min for the entire preparation.

Furthermore, the resulting suspension volume is very different between the two different processes. For the same silver nitrate concentration, the Lee–Meisel synthesis produces 250 mL of nanoparticle suspension, whereas the microwave-optimised method yields 15 mL of AgNps. Despite the smaller volume, it is generally adequate for a day of SERS analyses. Given that the suspension stability is not guaranteed over time, synthesising smaller volumes allows for freshly prepared substrates to be used. This approach minimises waste and saves time, in addition to offering better performances in SERS measurement.

## 3. Materials and Methods

### 3.1. Chemicals and Reagents

Silver nitrate and potassium chloride were provided by VWR (Pennsylvania, PA, USA). Trisodium citrate was purchased from Acros Organics (Geel, Belgium), while crystal violet was obtained by TCI Europe N.V. (Zwijndrecht, Belgium). All reactants presented an analytical grade. Solutions were prepared with Milli-Q water (18.2 MΩ·cm, Milli-Q Plus 185, Millipore, Burlington, MA, USA).

### 3.2. Silver Nanoparticle Synthesis

Taking into account the photochemical reduction susceptibility of silver, the nanoparticle syntheses were conducted under light-protected conditions.

The nanoparticle suspensions were prepared using a microwave (Biotage^®^ Initiator+ Microwave system with robot eight, Biotage, Uppsala, Sweden). In a vial adapted to the microwave (microwave reaction vials 10–20 mL, Biotage, Uppsala, Sweden), a magnetic bar and 15.0 mL of a silver nitrate solution of 1 × 10^−3^ M were added before the introduction of 200 µL of an aqueous solution of trisodium citrate where the concentration was determined by the investigation design. When the reducing solution was added in full, a delay of 40 s was respected to close the vial and start the reaction. The reaction parameters were defined according to the different experimental designs (see Section 3.5. Experimental designs). The temperature medium was directly measured inside the vial with the aid of an infrared sensor. The resulting suspension was cooled down at ambient temperature and conserved at 5 °C in a light-protected environment.

Silver nanoparticles were also synthesised using the Lee–Meisel protocol [20]. A 3-neck flask containing 250.0 mL of silver nitrate 1 × 10^−3^ M topped with a condenser was placed on a Drysyn heating bath (Asynt, Ely, UK) where the temperature was fixed at 180 °C. With the assistance of a dosing device (Dosimat, Metrohm AG, Herisau, Switzerland), 5 mL of a 0.04 M trisodium citrate solution was added at a rate of 5 mL/min when the solution boiled. After 60 min, the reaction was stopped, and the resulting suspension was cooled and stored at 5 °C.

### 3.3. AgNps Characterisation

The day after the synthesis, AgNps were characterised with different techniques. UV–visible spectra (Lambda 40, PerkinElmer, Waltham, MA, USA) were acquired from 200 to 800 nm. AgNps suspensions (returned to room temperature) were diluted 10 times with Milli-Q water and sonicated just before the analysis. The mean size and zeta potential were measured with a Malvern Panalytical Zetasizer Nano ZS (Malvern Panalytical, Malvern, UK) using dynamic light scattering (DLS) and an electrophoretic light scattering equipment with a laser wavelength of 632.8 nm. Before each measurement, 10 times diluted AgNps suspensions were sonicated.

The AgNps images were acquired by transmission electron microscopy (TEM) with a Jeol JEM-1400 TEM (Jeol, Tokyo, Japan) at 80 kV. A calibrated drop (11 µL) of suspension was deposited and dried on a copper-coated carbon grid (200 mesh, Jeol, Tokyo, Japan).

### 3.4. SERS Analyses

SERS acquisitions were made the day after the synthesis and after returning to room temperature, with a transmission Raman spectroscopy equipment (TRS 100, Agilent Technologies, Santa Clara, CA, USA). The laser wavelength was 830 nm with a spectral resolution for the entire range of less than 8 cm^−1^ and a spectral range from 50 to 2300 cm^−1^. The equipment configuration was 4M, which refers to the laser spot size (4 mm) and the lens collection optics (medium). The parameters were a laser power of 60 mW and an acquisition time of 5 s with 3 accumulations. For the investigation design, three successive measurements were recorded and averaged for each sample. For the optimisation design, the SERS signal was measured each minute during 10 min. The intensity over time was graphically reported to determine a stable period during which the mean intensity was calculated and presented as the result.

The SERS samples were prepared by mixing for 10 s with a vortex (Reax top, Heidolph, Schwabach, Germany) 400 µL of AgNps with 400 µL of crystal violet (0.75 × 10^−6^ M) in a vial (screw neck vial ND13 of 4 mL, VWR, Pennsylvania, PA, USA) before the addition of 100 µL of an aggregating agent. The resulting solution was vortexed for 10 s and eventually placed in the TRS 100. The aggregating agent (KCl) was studied at 3 concentrations (0.1, 0.3 and 0.5 M). Three independent samples were prepared for each AgNps suspension and each KCl concentration. 

The SERS spectra were processed with MatLab^®^ R2020b (The MathWorks, Natick, MA, USA) and PLS_Toolbox 8.9.2 (Eigenvector Research, Inc., Wenatchee, WA, USA). The applied preprocessing was a baseline correction with an automatic Whittaker filter (λ: 3 × 10^5^ and p: 0.01). The reported intensity is that of a characteristic band of the crystal violet (1170 cm^−1^) and was the average of the intensities at 1169, 1170 and 1171 cm^−1^ [42,43,44]. For each condition, the RSD was calculated on 3 replicates of 3 independent samples. Given that the citrate ions are replaced by the chloride ions of the aggregating agent, the SERS spectra obtained are characteristic of the crystal violet.

### 3.5. Experimental Designs

The elaboration and the interpretation of the design of experiments were performed with JMP^®^ Pro 15 software (SAS Institute, Cary, NC, USA).

The investigation design was an A-optimal design divided into 6 random blocks of 6 syntheses with 4 continuous variables. This design investigated the main effect, two-factor interaction and quadratic effects of 4 factors: the concentration of trisodium citrate, the time and the temperature of the reaction and the stirring speed. Given that the vial is sealed, an increase in pressure can lead to a rise in the boiling temperature of the aqueous solution. Based on preliminary tests, the design was constrained to exclude experiments at too low temperature and time combinations. To be included, the experiments must satisfy Equation (1).

The optimisation design was an I-optimal design investigating the main effects, two-factor interactions and quadratic effects of 3 factors (reaction time, reaction temperature and stirring speed) keeping the constraint described in Equation (1). The design was composed of 13 syntheses and was repeated 3 times on 3 different days. The process parameters (PPs) and the critical process parameters (CPPs) for these two experimental designs are presented in Table 1.

## 4. Conclusions

To conclude, this study aimed to propose a new strategy based on a Quality by Design approach to robustly optimise a synthesis protocol of AgNps, being low cost, rapid and easy to implement, to improve the Np properties of homogeneity and their repeatability from batch to batch. This robust optimisation could also lead to an improvement in the protocol reproducibility from laboratory to laboratory. A chemical reduction method inspired by the widely used process created by Lee and Meisel associated with microwave irradiation was selected. With the objective of efficiently optimising the synthesis process, the Quality by Design strategy was used. Based on an Ishikawa diagram, the criticality of 4 process parameters was evaluated. The CQAs consisted of a high SERS signal intensity with the lowest RSD possible. An investigation design showed that the reaction time, temperature and stirring speed had an impact on the AgNps formation and needed to be optimised. Two interesting areas were calculated: one for higher temperature and lower reaction times and another for lower temperature and higher reaction times. The faster conditions were retained for the optimisation design. A method operable design region (MODR) was identified where signal intensities were higher than 10,000 counts and the intra- and intra- + inter-synthesis variabilities were lower than 15% and 20%, respectively. A set of optimal conditions (reaction time: 3.36 min, reaction temperature: 130 °C, stirring rate: 600 rpm) was selected in this MODR to validate the conclusions. These optimal conditions were used to realise six syntheses. These syntheses were fully characterised and presented results very close to each other. Concerning the SERS results, a variability from 7 to 15% within batches was observed, while the variability from one batch to another was 9%. These results conformed to the expected CQAs. Furthermore, a comparison was made between the performance of the classical Lee–Meisel synthesis and the microwave-optimised process. While the intra-synthesis performances are comparable for both synthesis routes, the intra- and inter-synthesis variabilities were reduced significantly when using the microwave method. In conclusion, the QbD strategy was successfully used to robustly optimise the AgNps synthesis by a microwave reduction with the acquisition of maximum information including interactions with a minimum of experiments. This strategy could be implemented on other synthesis protocols’ optimisation of SERS substrates, which might improve the inter-laboratory reproducibility. All these aspects could enhance the quantitative perspectives of SERS in various domains, notably the pharmaceutical and biomedical fields. Indeed, to measure a repeatable signal, the use of repeatable SERS substrates is essential.

## Figures and Tables

**Table 1:** Selected variables for the experimental designs.

Variable	Investigation Design (PPs)	Optimisation Design (CPPs)
Citrate concentration (M)	0.03–0.08	Not included
Reaction time (min)	1–20	1–8
Reaction temperature (°C)	100–170	130–170
Stirring speed (rpm)	300–660	300–660

**Table 2:** Optimisation design characteristics and results.

Time (min)	Temperature (° C)	Stirring Speed (rpm)	Mean Intensity (Counts)	Intra- Synthesis RSD (%)	Intra- + Inter-Synthesis RSD (%)
3.36	130	300	37,627.1	19.6	35.5
4.48	151	480	14,389.9	10.1	35.2
8	150	300	448.1	16.1	28.1
2.24	170	300	203.6	13.8	26.8
5	170	660	219.4	12.2	13.4
4.48	151	480	9061.3	13.8	74.4
1	170	660	12,643.4	12.3	88.1
8	150	660	663.5	11.3	68.0
3.36	130	660	41,688	13.2	13.2
4.48	151	480	18,591.7	29.2	130.3
8	130	480	43,542.1	15.6	16.3
1	150	420	27,971.5	12.7	60.9
8	170	480	238.8	14.6	19.3

## Data Availability

Data available on request.

## Supplementary Materials

The following supporting information can be downloaded at: https://www.mdpi.com/article/10.3390/molecules29143442/s1, Table S1: Abbreviations of the Quality by Design concept and some explanations of each term; Table S2: Synthesis conditions and results for the investigation design; Figure S1: SERS spectra to (a) compare different syntheses of the investigation design with the optimal conditions, (b) show the 6 replicates of optimal conditions and (c) compare mean spectra (n = 18) for optimal conditions using microwave and for AgNps synthesised according to the Lee–Meisel protocol; Figure S2: Pictures obtained by transmission electron microscopy for silver nanoparticles synthesized during the first replicate of the optimisation design; Figure S3: Simulated conditions with the regression models of the optimisation design. The dots are coloured based on the function of the log transformed SERS signal intensity. Yellow conditions denote high signal intensity, and blue conditions denote low signal intensity; Figure S4: Simulated conditions with the regression models of the optimisation design. The dots are coloured based on the function of the log transformed intra-synthesis RSD%. Yellow conditions denote low intra-synthesis variability, and blue conditions denote high intra-synthesis variability; Figure S5: Simulated conditions with the regression models of the optimisation design. The dots are coloured based on the function of the log transformed intra- + inter-synthesis RSD%. Yellow conditions denote low intra- + inter-synthesis variability, and blue conditions denote high intra- + inter-synthesis variability; Figure S6: Pictures obtained by transmission electron microscopy for some silver nanoparticles synthesised according to the Lee–Meisel protocol; Table S3: Variation coefficients (RSD, %, n = 9) of the SERS measurements for 4 different concentrations of crystal violet for two synthesis processes; Figure S7: UV–visible spectra for 6 syntheses of AgNps synthesised by microwave irradiation (blue) and for 5 syntheses obtained by the Lee–Meisel protocol (pink).
