# Supplementary material for: Optimisation of a Microwave Synthesis of Silver Nanoparticles by a Quality by Design Approach to Improve SERS Analytical Performances
Source: Molecules. 2024 Jul 22;29(14):3442. doi: 10.3390/molecules29143442 (PMC11280077; doi:10.3390/molecules29143442)
Supplement: Supplementary file 1 [file molecules-29-03442-s001.zip › molecules-3039803-supplementary.pdf]

Optimisation of a microwave synthesis of silver nanoparticles by a quality by design approach to improve SERS analytical performances

Julie Horne [1,*], Pierre Beckers [1], Pierre-Yves Sacré [2,*], Charlotte De Bleye [1], Pierre Francotte [3], Nicolas Thelen [4], Philippe Hubert [1], Eric Ziemons [1,†] and Cédric Hubert [1,†]

[1] Laboratory of Pharmaceutical Analytical Chemistry, Department of Pharmacy, CIRM, ViBra-Sante Hub, University of Liege (ULiege), 4000 Liege, Belgium
[2] Research Support Unit in Chemometrics, Department of Pharmacy, CIRM, University of Liege (ULiege), 4000 Liege, Belgium
[3] Laboratory of Medicinal Chemistry, Department of Pharmacy, CIRM, University of Liege (ULiege), 4000 Liege, Belgium
[4] GIGA-Neurosciences, Cell Biology, University of Liege (ULiege), 4000 Liege, Belgium
***** Correspondence: julie.horne@uliege.be (J.H.); pysacre@uliege.be (P.-Y.S.)
† These authors contributed equally to this work.

Supplementary data

Table S1. Abbreviations of the Quality by Design concept associated to some explanations of each term.

Abbreviation	Meaning	Definition
QbD	Quality by Design	Strategy to robustly optimise a process by realising successive designs of experiment
QTPP	Quality target product profile	Objective(s) of the study
CQA	Critical quality attribute	Criterion(a) to evaluate the response quality
PP	Process parameter	Parameters giving the response requiring by the CQA
CPP	Critical process parameter	PP presenting a critical impact on the CQA
DoE	Design of experiment	Manipulation to realise to obtain a maximum of information with a minimum of experiment
MODR	Method operable design region	Modelisation of the different parameters showing the probability to achieve the goal for each parameter's combination

Table S2. Syntheses conditions and results for the investigation design.

Random Block	Temperature (°C)	Time (min)	Citrate concentration (M)	Stirring speed (rpm)	SERS intensity (counts)	SERS RSD (%)	Selected KCl concentration (M)
1	100	20	0.03	480	35811.0	35.6	0.3
1	170	1	0.03	300	5713.8	27.1	0.5
1	100	20	0.03	480	10912.6	8.1	0.5
1	142	12.8	0.055	660	301.2	2.0	0.1
1	170	1	0.03	300	4888.8	21.6	0.5
1	142	12.8	0.055	660	1816.2	23.4	0.5
2	170	20	0.08	660	264.6	3.1	0.1
2	128	11.9	0.08	480	55458.0	4.8	0.3
2	170	1	0.08	660	51815.9	3.9	0.3
2	170	1	0.08	660	52549.1	7.4	0.3
2	170	20	0.08	660	183.6	3.2	0.3
2	128	11.9	0.08	480	41454.9	20.6	0.3
3	170	20	0.03	660	221.6	3.2	0.3
3	170	20	0.03	660	232.1	5.6	0.3
3	100	7.5	0.055	480	22087.3	20.5	0.3
3	100	7.5	0.055	480	20582.0	31.0	0.3
3	135	14.9	0.03	300	14224.0	10.9	0.5
3	135	14.9	0.03	300	399.5	4.0	0.3
4	170	10.5	0.08	300	184.4	5.0	0.3
4	142	2	0.055	480	32202.3	27.5	0.3
4	142	2	0.055	480	33836.0	30.2	0.3
4	100	20	0.08	300	32330.7	10.5	0.3
4	170	10.5	0.08	300	194.3	2.9	0.3
4	100	20	0.08	300	41457.1	4.5	0.3

5	100	7.5	0.08	660	4387.4	15.8	0.5
5	100	7.5	0.08	660	4584.7	33.5	0.5
5	170	8.6	0.055	480	169.0	9.2	0.3
5	142	20	0.08	480	207.6	2.9	0.3
5	150	1	0.08	300	28160.7	14.6	0.3
5	170	8.6	0.055	480	213.0	1.5	0.5
6	100	20	0.055	660	7786.6	7.0	0.5
6	150	1	0.03	660	4408.2	16.9	0.3
6	150	1	0.03	660	18563.5	10.1	0.5
6	170	8.6	0.03	500	353.6	1.3	0.1
6	170	20	0.055	300	191.5	4.8	0.5
6	100	7.5	0.03	300	7283.2	26.2	0.3

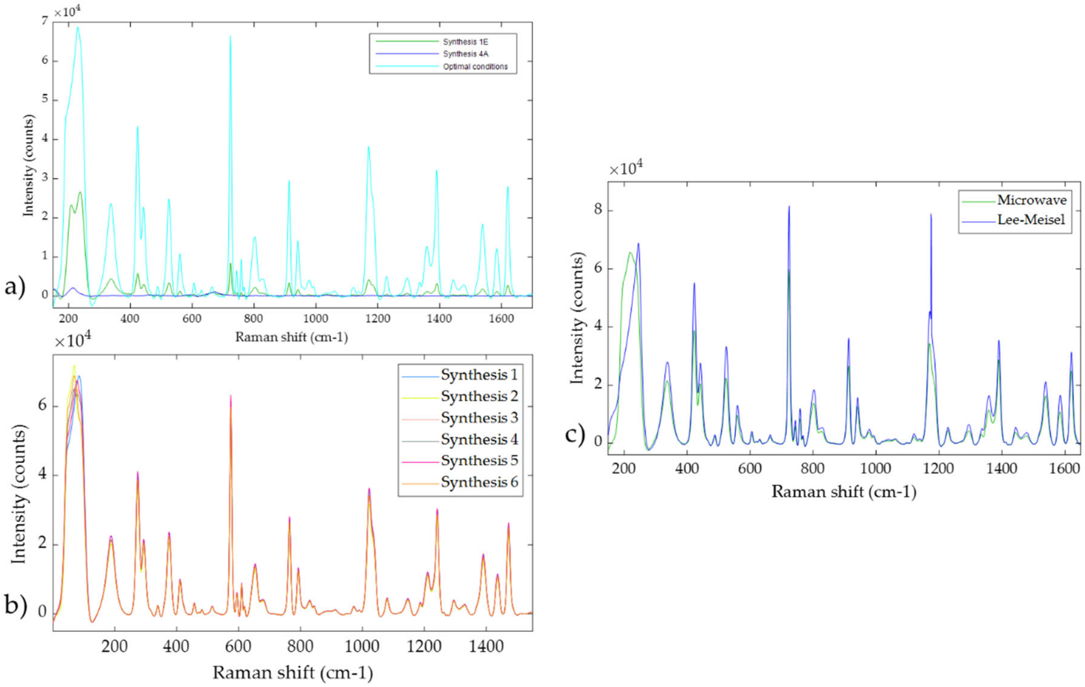

Figure S1. SERS spectra to (a) compare different syntheses of the investigation design with the optimal conditions, (b) show the 6 replicates of optimal conditions and (c) compare mean spectra (n=18) for optimal conditions using microwave and for AgNps synthesised according to the Lee-Meisel protocol.

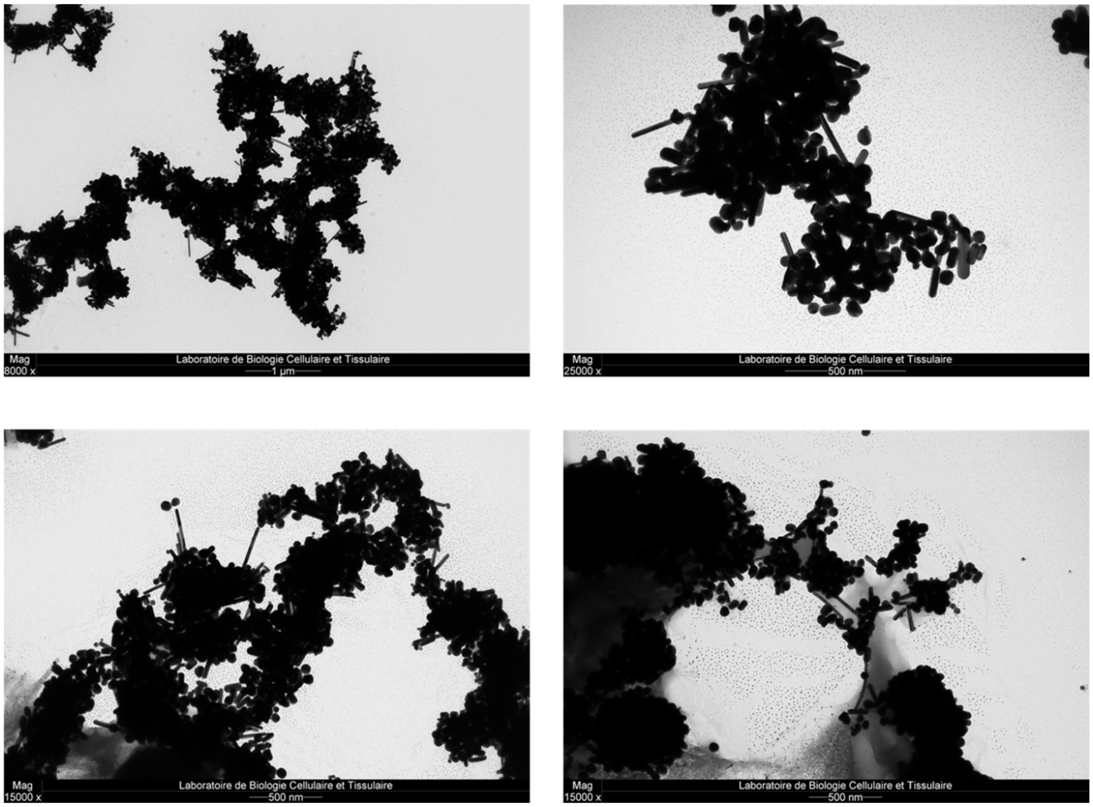

Figure S2. Pictures obtained by transmission electron microscopy for silver nanoparticles synthesized during the first replicate of the optimisation design.

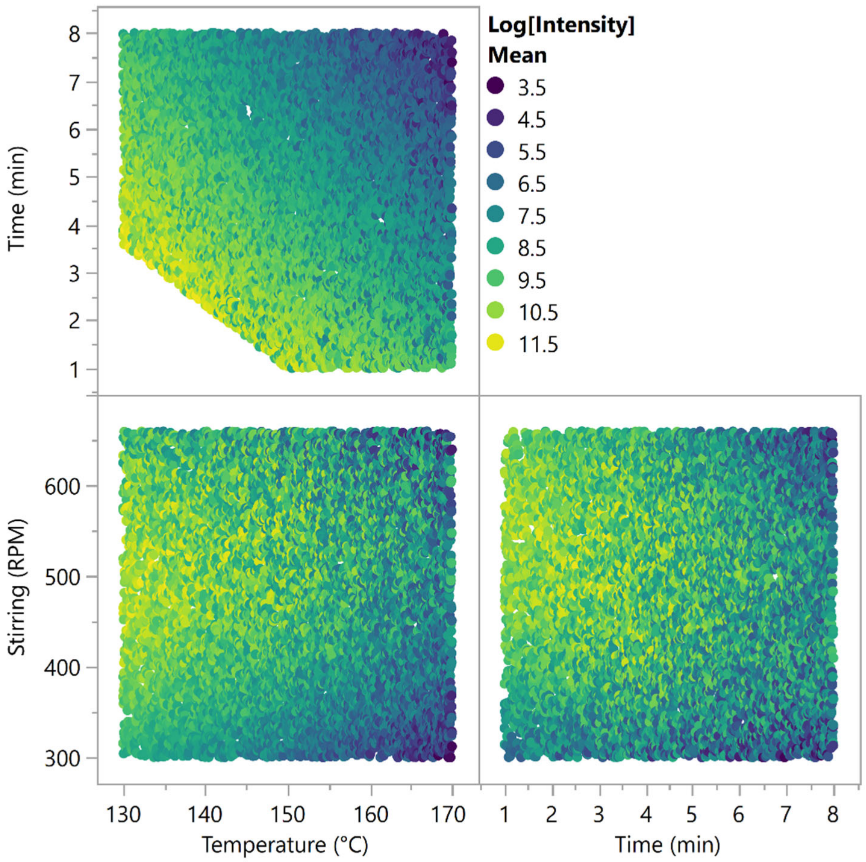

Figure S3. Simulated conditions with the regression models of the optimisation design. The dots are colored in function of the log transformed SERS signal intensity. Yellow conditions denote high signal intensity, blue conditions denote low signal intensity.

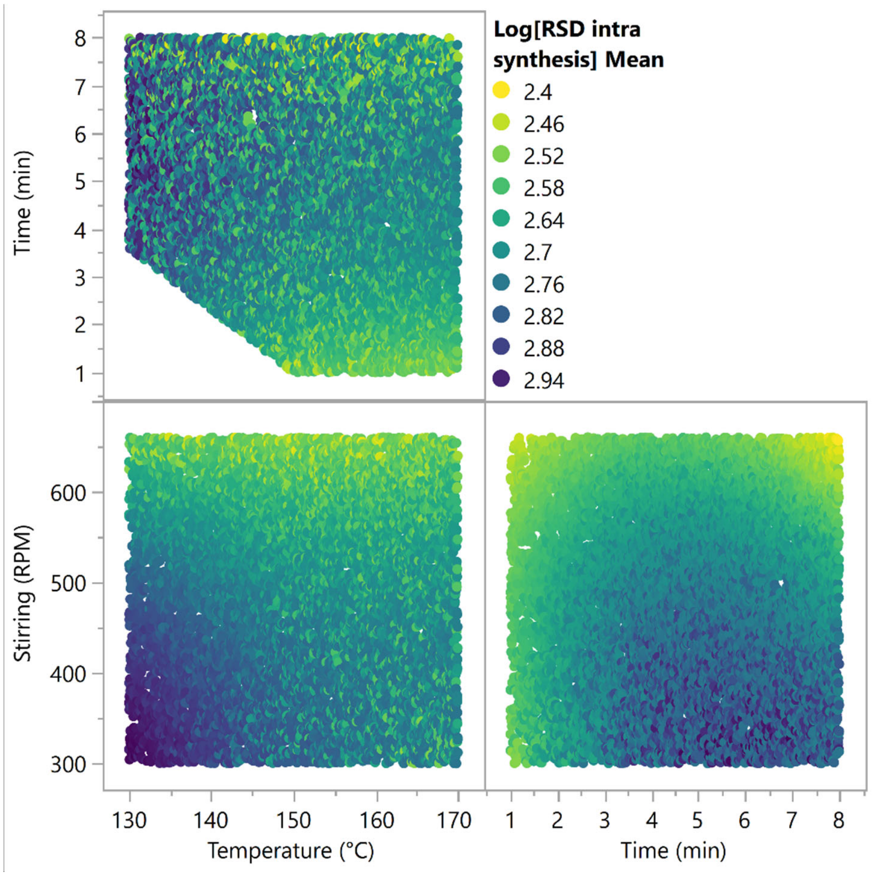

Figure S4. Simulated conditions with the regression models of the optimisation design. The dots are colored in function of the log transformed intra-synthesis RSD. Yellow conditions denote low intra-synthesis variability, blue conditions denote high intra-synthesis variability.

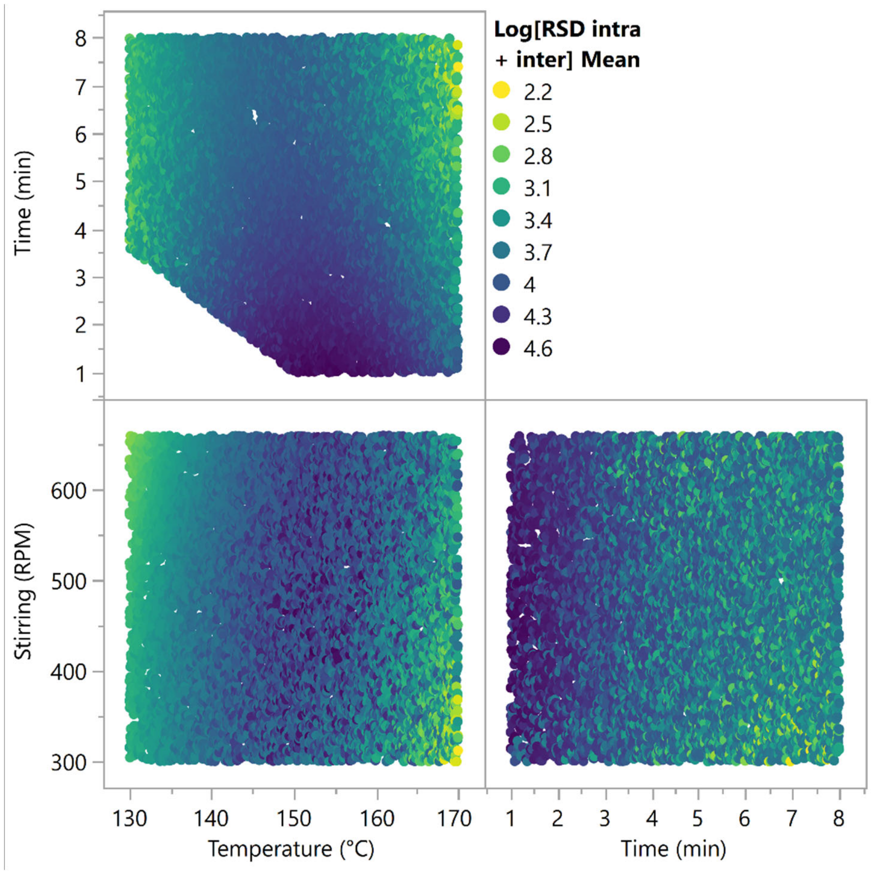

Figure S5. Simulated conditions with the regression models of the optimisation design. The dots are colored in function of the log transformed intra + inter synthesis RSD. Yellow conditions denote low intra + inter-synthesis variability, blue conditions denote high intra + inter-synthesis variability.

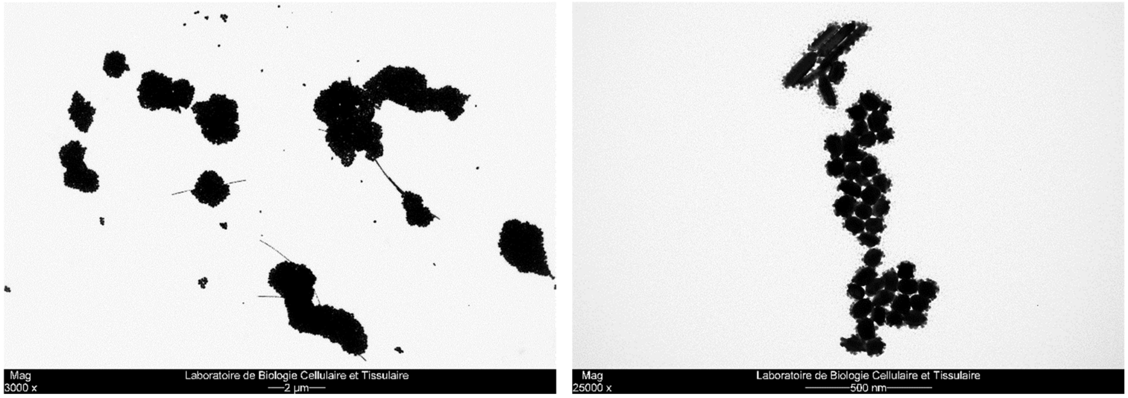

Figure S6. Picture obtained by transmission electron microscopy for silver nanoparticles synthesised according to the Lee-Meisel protocol.

Table S3. Variation coefficients (RSD, %, n=9) for SERS measurements for 4 different concentrations of crystal violet for two synthesis processes.

RSD (%, n= 9)	Concentration (x10^{-6} M)			
	0.50	0.75	0.80	1.00
Lee-Meisel	3.74	6.83	11.14	6.31
Microwave	4.14	3.06	4.05	2.26

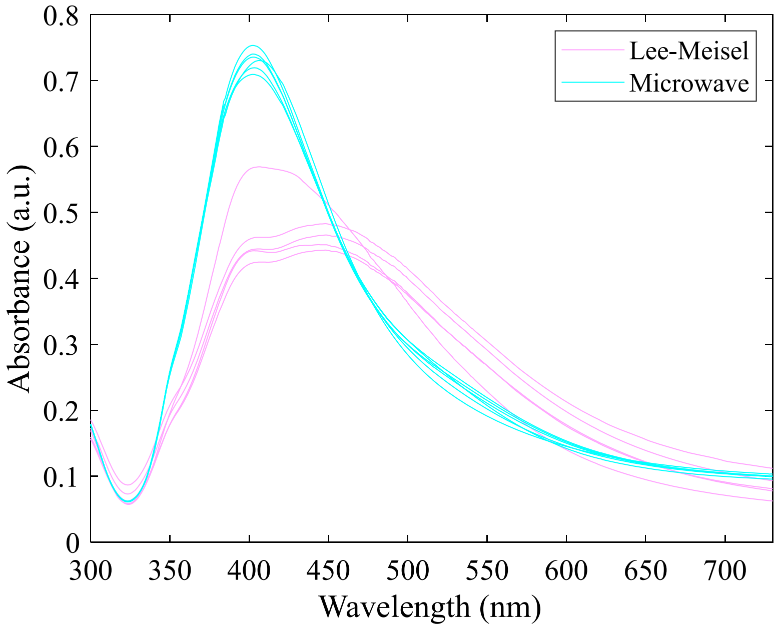

Figure S7. UV-visible spectra for 6 syntheses of AgNps synthesised by microwave irradiation (blue) and for 5 syntheses obtained by the Lee-Meisel protocol (pink)
